# Improved identification of tumors in 18F-FDG-PET examination by normalizing the standard uptake in the liver based on blood test data

**DOI:** 10.1007/s11548-023-03044-4

**Published:** 2024-01-05

**Authors:** Md Ashraful Alam, Shouhei Hanaoka, Yukihiro Nomura, Tomohiro Kikuchi, Takahiro Nakao, Tomomi Takenaga, Naoto Hayashi, Takeharu Yoshikawa, Osamu Abe

**Affiliations:** 1 Department of Computational Diagnostic Radiology and Preventive Medicine, The University of Tokyo Hospital, 7-3-1 Hongo, Bunkyo-ku, Tokyo, 113-8655 Japan; 2 Department of Radiology, The University of Tokyo Hospital, 7-3-1 Hongo, Bunkyo-ku, Tokyo, Japan; 3 Center for Frontier Medical Engineering, Chiba University, 1-33 Yayoicho, Inage-ku, Chiba, Japan; 4 Department of Radiology, School of Medicine, Jichi Medical University, 3311-1 Yakushiji, Shimotsuke, Tochigi, 329-0498 Japan; 5 Radiology and Biomedical Engineering, Graduate School of Medicine, University of Tokyo, Tokyo, Japan

**Keywords:** ^18^F-fluorodeoxyglucose positron-emission tomography, Standardized uptake value, Z-score, Liver tumors

## Abstract

**Purpose:**

Standardized uptake values (SUVs) derived from ^18^F-fluoro-2-deoxy-D-glucose positron emission tomography/computed tomography are a crucial parameter for identifying tumors or abnormalities in an organ. Moreover, exploring ways to improve the identification of tumors or abnormalities using a statistical measurement tool is important in clinical research. Therefore, we developed a fully automatic method to create a personally normalized Z-score map of the liver SUV.

**Methods:**

The normalized Z-score map for each patient was created using the SUV mean and standard deviation estimated from blood-test-derived variables, such as alanine aminotransferase and aspartate aminotransferase, as well as other demographic information. This was performed using the least absolute shrinkage and selection operator (LASSO)-based estimation formula. We also used receiver operating characteristic (ROC) to analyze the results of people with and without hepatic tumors and compared them to the ROC curve of normal SUV.

**Results:**

A total of 7757 people were selected for this study. Of these, 7744 were healthy, while 13 had abnormalities. The area under the ROC curve results indicated that the anomaly detection approach (0.91) outperformed only the maximum SUV (0.89). To build the LASSO regression, sets of covariates, including sex, weight, body mass index, blood glucose level, triglyceride, total cholesterol, γ-glutamyl transpeptidase, total protein, creatinine, insulin, albumin, and cholinesterase, were used to determine the SUV mean, whereas weight was used to determine the SUV standard deviation.

**Conclusion:**

The Z-score normalizes the mean and standard deviation. It is effective in ROC curve analysis and increases the clarity of the abnormality. This normalization is a key technique for effective measurement of maximum glucose consumption by tumors in the liver.

## Introduction

Medical imaging is subjectively or quantitatively evaluated in clinical practice [1]. Positron emission tomography (PET)/computed tomography (CT) is a widely used minimally invasive imaging modality for evaluating various neoplasms and other diseases of the human body [2]. The radioactive tracer ^18^F fluro-2- deoxy-D-glucose (^18^F-FDG) is used in combination with PET for diagnostic purposes to identify tissues with altered glucose metabolism [3]. Standardized uptake value (SUV) is a semi-quantitative measurement of ^18^F-FDG tracer uptake in tissues [4]. The SUV is influenced by several variables, including weight, sex, body mass index (BMI), plasma glucose level, duration of uptake phase, partial-volume effects, and recovery coefficient [1, 4].

The liver is a useful organ for evaluating FDG uptake in quality control [5], therapy assessment [6], and prognosis [7]. Owing to its large size, it is clearly seen on FDG-PET images. The liver may exhibit increased FDG uptake even in the absence of malignancy. Various background SUVs can contribute to the failure to detect liver tumors or cancer. In addition, a genuine hepatic lesion with moderately elevated uptake may be unnoticed. Several clinical variables have been reported to have an impact on hepatic FDG absorption [8]. Therefore, it is necessary to normalize them for easier detection of tumors by physicians, for example, by simply thresholding the “normalized” SUV at its maximum.

Clinical variables for the diagnosis and treatment of liver disease are often obtained from blood tests, commonly referred to as liver function tests. Abnormal blood test results are often the first sign of liver diseases, such as cirrhosis, and are sometimes used as a guide for PET/CT imaging of the abdomen. Serum indicators, such as aspartate aminotransferase (AST), alanine aminotransferase (ALT), alkaline phosphatase (ALP), bilirubin, and albumin, are commonly used to screen for liver abnormalities [9]. Moreover, age [10], blood glucose level [11], and BMI [12] have all been shown to affect liver FDG absorption. Abdominal CT is the first imaging modality used when imaging is necessary to examine anomalies [9]. We searched PubMed using the keywords “Diagnosis of FDG/PET,” “blood test results,” and “Z-score.” No previously published articles related to the objectives of the present study were identified.

PET/CT-derived SUVs of healthy background liver tissues are often used as a reference to characterize anomalies and assess a tumor’s response to treatment. Therefore, we aimed to enhance the diagnostic ability of FDG-PET by normalizing the liver SUV in each patient. We estimated the average SUV (and standard deviation) of the liver from various non-image-derived variables using machine learning-based methods. We also established a fully automatic method to create a personally normalized Z-score map of the liver SUV. The Z-score method is known as the standard score or standardized value method. It is a statistical technique used to assess how far a particular data point is from the mean of a dataset, and it is measured in terms of statistical deviations. The Z-score helps to understand how extreme or unusual a data point is within a distribution. Moreover, we aimed to establish a least absolute shrinkage and selection operator (LASSO)-based estimation formula for normalizing individual hepatic SUV maps. This regression method is primarily used for predictive modeling and regression analysis. The LASSO regression is commonly used in situations where there are many features but not all are important for the prediction. Moreover, this model helps balance model complexity and predictive accuracy by encouraging sparse models and automatically selecting features in the presence of high-dimensional data.

Using the proposed method, the SUV map can be replaced with a normalized Z-score map for daily image interpretation by physicians. Hopefully, this will help detect and diagnose hepatic masses more accurately and in a more standardized manner. We also analyzed the receiver operating characteristic (ROC) curve results of subjects with and without hepatic tumors and compared them with the ROC curve of normal SUV.

## Materials and methods

### Study design and dataset

This retrospective study was approved by our institutional review board. The study population was defined as adults who visited our hospital for a whole-body medical screening program between November 2006 and November 2017. All the participants provided written explicit consent for the use of their medical images, blood samples, and other demographic data. For medical image screening, PET/CT was performed using single-type scanners (Discovery ST Elite, GE Healthcare, Waukesha, WI, USA). CT images were obtained using the following parameters: field of view (FOV), 500 mm; matrix size, 512 × 512; voxel size, 0.98 mm × 0.98 mm × 1.25 mm. PET images were obtained using the following parameters: FOV, 700 mm; matrix size, 128 × 128; voxel size, 5.47 mm × 5.47 mm × 3.25 mm. In each instance, blood tests were performed on the same day as the PET/CT scans. All PET/CT images were reviewed twice throughout the screening program. The final diagnosis was reached after discussions between two radiologists who independently read identical PET/CT images. No intravenous contrast medium was used for the CT.

**Fig. 1:**
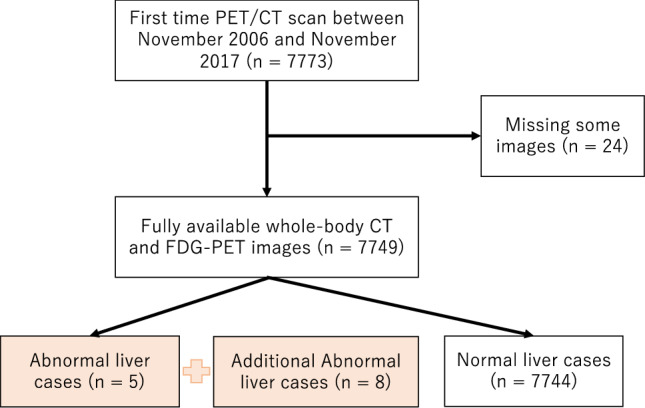
Flowchart of study inclusion

Figure 1 shows the inclusion flowchart of the subjects. The first time PET/CT scans were performed; there were 7,773 cases over the aforementioned time frame. Among these, some images were missing in 24 cases; therefore, these cases were excluded. Consequently, 7,749 fully available whole-body CT and FDG-PET volumes were obtained. A total of 7744 cases were identified as normal, and five cases were identified as abnormal. Local tumors and diffuse liver diseases (e.g., cirrhosis) were not included in the abnormal dataset. However, localized mass-like lesions were the focus of this study. Since there were very few abnormal cases in the first-time visit dataset, we decided to include additional abnormal cases from our hospital dataset. Therefore, eight more abnormal cases were included. Finally, we included 7744 normal and 13 abnormal cases in our analysis.

We also collected the following variables from the selected (PET/CT) subjects: height, weight, BMI, age, amylase, high-density lipoprotein (HDL) cholesterol, low-density lipoprotein (LDL) cholesterol, platelet count, total cholesterol, total bilirubin, albumin, total protein, cholinesterase, ALP, triglyceride, ALT, γ-glutamyl transpeptidase (γ-GTP), AST, insulin, HbA1c, blood glucose, creatinine, and Brinkman index (this index is defined as the number of cigarettes smoked per day multiplied by years of smoking) [13]. All tests, including blood tests, were performed on the same day as the PET/CT examinations.

### Image processing

We performed whole-abdominal CT image segmentation to obtain the labels of each abdominal organ, particularly the liver. We used a self-supervised pipeline for 3D segmentation, as described by NVIDIA. A detailed description of this process has been provided in a previous study [14]. The CT images were cropped to 512 × 512 × 400 voxels from the center point. The PET images were resized to fit the voxel size of the whole-body CT and cropped to 512 × 512 × 400 voxels using the same location information. Segmentation of the liver region was performed using only the CT volume, and not the PET volume. Using the segmentation map, the CT value (mean of the liver segmentation map) from CT images, and the mean SUV (mean of the segmentation map) and standard deviation (standard deviation of the segmentation map) from PET images were calculated.

### Statistical analysis

The summary statistics of the variables are presented as the mean ± standard deviation. To compare between-group differences between the training and test groups, we used the Student’s *t* test or Mann–Whitney U test, as appropriate, for continuous variables. Statistical significance was defined as *p* < 0.05. Correlations between variables were calculated using Spearman’s correlation analysis.

We calculated a patient-wise normalized SUV (Z-score) map using the estimated SUV mean and standard deviation (estimated using blood-test-derived variables and demographic data).

We created a model using the LASSO regression of the SUV mean and standard deviation with the selected covariates using the training dataset.

The LASSO regression drops off coefficients in the regression by forcing a penalty on the regularization term called “the sum of the absolute value of the coefficients” [15]. The objective function of the LASSO is expressed as:

1 $${min}_{\beta \in {\mathbb{R}}^{p}}\left\{{\Vert Y-X\beta \Vert }_{2}^{2}+\lambda \sum_{j=1}^{p}\left|{\beta }_{j}\right|\right\}$$

where $\beta$ is a vector whose nonzero elements represent the selected variables, $X$ is the data matrix (i.e., independent variables), and $Y$ is the vector of dependent variables (either the estimated mean or standard deviation of liver SUVs in this study) of all training subjects. Here $\lambda$ is a tuning parameter that controls the strength of regularization. When the $\lambda$ value is large, more regression coefficients $\beta$ drop off. To obtain the best model, $\lambda$ plays a key role. In this study, we decided that the number of selected variables should not be too large (approximately 10 variables), considering their usefulness in daily clinical routines. Finally, we applied the model to the test dataset and abnormal cases and calculated the estimated SUV mean and standard deviation for each subject.

Therefore, we built a Z-score map using the following equation:

2 $${Z}_{i}= \frac{{y}_{i}-E(Y)}{\sqrt{Var(Y)}}$$

where $$i$$ denotes each pixel, $${Z}_{i}$$ indicates the Z-score of the $$i$$-th pixel of the actual PET slice, *E(Y* $$)$$ is the estimated mean SUV, and *SD(Y* $$)$$ is the estimated SUV standard deviation. Therefore, the Z-score represents the difference in SUV from the mean in units of the standard deviation. Abnormal FDG uptake is indicated by a higher absolute Z-score. Note that the SUV of most tumor lesions is higher than that of normal liver tissue. Therefore, in this study, we focused on focal positive alterations in *Z*-score within each liver after personal normalization. Our LASSO regression model reflects and normalizes the “background” liver SUV because it was trained using only healthy subjects. We hypothesized that this model would identify abnormal tumor lesions in the liver.

To assess the ability of the patient-wise Z-score to distinguish between normal and abnormal tissues, we conducted a ROC analysis. Abnormalities in the SUV and $$Z$$-score were represented by the maximum SUV ($${SUV}_{max})$$ and $$Z$$-score (Z-score_max_) within the automatically segmented liver region of each subject. In this study, we hypothesized that a tumor lesion in the liver would have a higher value/score than the background (i.e., normal liver tissue). We compared the tumor detection abilities of the SUV_max_ and Z-score_max_ using patient-wise ROC analysis. Either the $${{\text{SUV}}}_{{\text{max}}}$$ or Z-score_max_ was thresholded using various thresholds, and patient-wise sensitivities and specificities were plotted as ROC curves. Finally, the areas under the ROC curves (AUROCs) of both $${{\text{SUV}}}_{{\text{max}}}$$ and Z-score_max_ were calculated and compared. We used twofold cross-validation in the LASSO regression.

All statistical analyses were performed using R software version 4.2.1. To perform LASSO regression, we used the *glmnet* package in the R software.

## Results

A total of 7744 patients were selected for further analysis from 7773 first time visited patients between the time duration of November 2006 and November 2017. We present the selection procedure in Fig. 1. After selection of the sample, we randomly divided the dataset into training set (n = 3872) and test set (n = 3872). We calculated the mean and standard deviation as a basic statistical result of demographic characteristics and blood test data of the training dataset, test dataset, and total cases (n = 7744). For more details, see Table 1. A true difference was found in mean result between the training and test datasets. More specifically, ALT and AST show a significant true difference in mean between the training and test datasets.

**Table 1:** Patient characteristics of demographic and blood sample data for all samples, training sets, and test sets, presented as mean and standard deviation

Variables	Training set (n = 3872)	Test set (n = 3872)	All (n = 7744)	*p*-value
Age	55.53 (10.65)	55.92 (10.62)	55.73 (10.63)	0.11
Height	164.76 (8.72)	164.72 (9.17)	164.74 (8.95)	0.87
Weight	65.18 (13.14)	65.18 (13.14)	65.18 (13.14)	0.99
BMI	23.85 (3.54)	23.86 (3.6)	23.85 (3.57)	0.96
Brinkman index	323.42 (445.41)	328.26 (450.68)	325.84 (448.03)	0.63
SUV mean	2.06 (0.27)	2.06 (0.27)	2.06 (0.27)	0.34
SUV SD	0.42 (0.15)	0.42 (0.17)	0.42 (0.16)	0.87
Amylase	74.32 (31.25)	73.52 (33.43)	73.92 (32.36)	0.28
HDL cholesterol	61.69 (16.94)	61.7 (17.37)	61.69 (17.15)	0.98
LDL cholesterol	124.87 (31.56)	124.14 (31.83)	124.5 (31.7)	0.31
Total cholesterol	203.07 (34.32)	202.46 (34.37)	202.76 (34.34)	0.44
Total bilirubin	0.9 (0.33)	0.89 (0.32)	0.9 (0.33)	0.31
Total protein	6.89 (0.39)	6.89 (0.39)	6.89 (0.39)	0.75
Platelet	23.21 (5.42)	23.26 (5.63)	23.23 (5.53)	0.66
Albumin	4.14 (0.26)	4.13 (0.26)	4.14 (0.26)	0.53
Cholinesterase	333.6 (69.22)	334.43 (69.3)	334.02 (69.26)	0.60
Alkaline phosphate	190.93 (69.95)	189.94 (58.94)	190.44 (64.68)	0.50
Triglyceride	120.11 (90.9)	121.66 (95.12)	120.89 (93.03)	0.46
Alanine Transaminase	25.32 (25.23)	23.89 (17.87)	24.61 (21.88)	0.004*
Aspartate Aminotransferase	23.13 (14.97)	22.47 (11.4)	22.8 (13.3)	0.03*
γ-Glutamyl Transpeptidase	48.15 (62.17)	46.77 (59.91)	47.46 (61.05)	0.32
Insulin	5.81 (7.2)	5.61 (5.17)	5.71 (6.27)	0.16
HbA1C	5.8 (0.79)	5.79 (0.79)	5.8 (0.79)	0.87
Blood glucose	96.79 (17.8)	96.57 (18.46)	96.68 (18.14)	0.59
Creatinine	0.76 (0.24)	0.77 (0.29)	0.76 (0.27)	0.21

*True difference in mean was not equal to zero between the training and test datasets

**Fig. 2:**
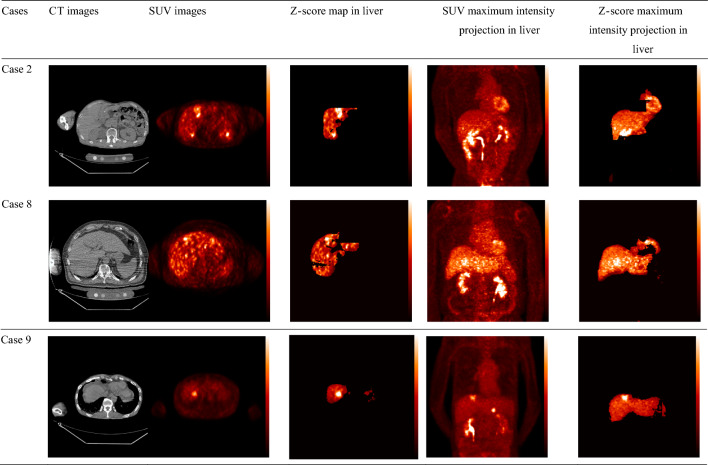
A visual representation of the standardized uptake value and Z-score in positron emission tomography/computed tomography images to understand the differences between them. We present three cases as examples

We also investigated the focal lesions with local SUV elevation in the abnormal cases. We present the pathologies of all 13 abnormal cases in Table 2. These pathological diagnoses were confirmed by a hospital physician. Among the abnormal cases, hepatocellular carcinoma was found in 5 cases. In four cases, we found multiple number of tumors. Besides this tabular pathological information, we show a pictorial representation (Fig. 2) of the Z-score maps obtained using our anomaly-detection method. We present three cases as an example out of 13 abnormal cases. In this figure, the original images (PET/CT) are presented in the first and second columns. The Z-score maps and SUV maximum intensity projection figures are presented in the third and fourth columns. And in the last column, Z-score maximum intensity projection in liver was presented. This pictorial presentation of Z-score and SUV itself result magnifies the improved identification method of Z-score for abnormalities detection comparing the SUV result.

**Table 2:** List of abnormal cases

Case number	Pathology	Tumor numbers
Case 1	Sigmoid colon cancer liver metastases	2
Case 2	Inflammatory pseudotumor, suspected	1
Case 3	Hepatocellular carcinoma	1
Case 4	Metastatic cancer of unknown origin	1
Case 5	Hepatocellular carcinoma	1
Case 6	Combined intrahepatic cholangiocarcinoma and hepatocellular carcinoma	1
Case 7	Hepatocellular carcinoma	1
Case 8	Hepatocellular carcinoma	1
Case 9	Hepatocellular carcinoma	> 2
Case 10	Mammary cancer liver metastases	Multiple
Case 11	Gastric cancer liver metastases	Multiple
Case 12	Pancreatic neuroendocrine tumor liver metastases	Multiple
Case 13	Colon cancer liver metastases	Multiple

**Fig. 3:**
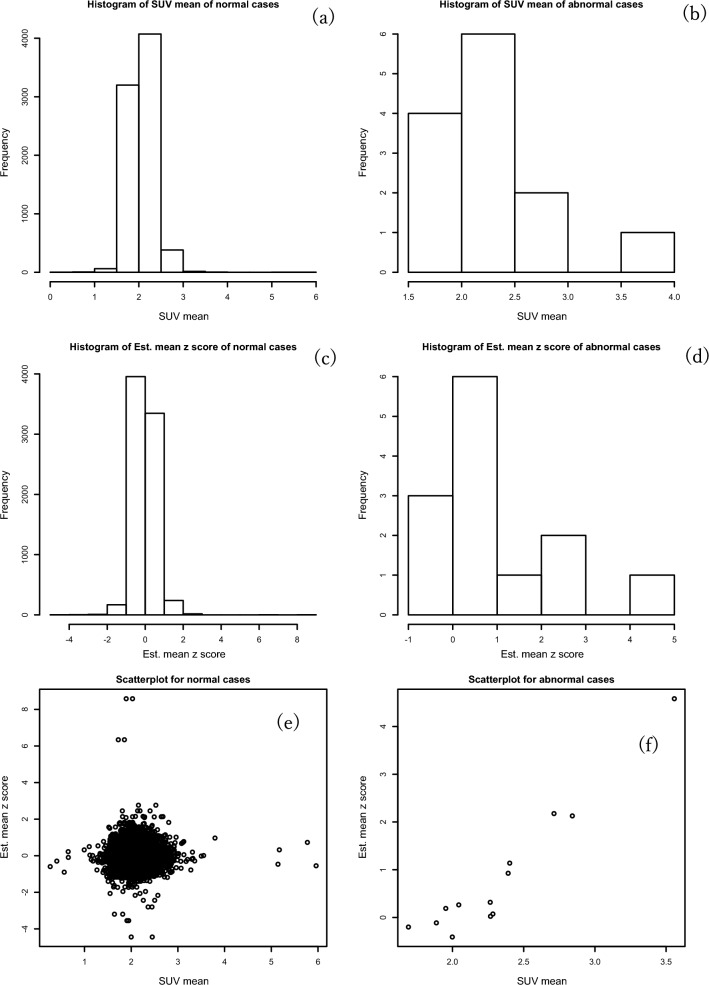
Summary plots of the mean standardized uptake value (SUV) and estimated mean Z-score: (**a**) histogram of the mean SUV of normal cases, (**b**) histogram of the mean SUV of abnormal cases, (**c**) histogram of the estimated mean Z-score by the least absolute shrinkage and selection operator (LASSO) regression of normal cases, (**d**) histogram of the estimated mean Z-score by the LASSO regression of abnormal cases, (**e**) scatterplot of normal cases using the mean SUV and estimated mean Z-score, (**f**) scatterplot of abnormal cases using the mean SUV and estimated mean Z-score

**Fig. 4:**
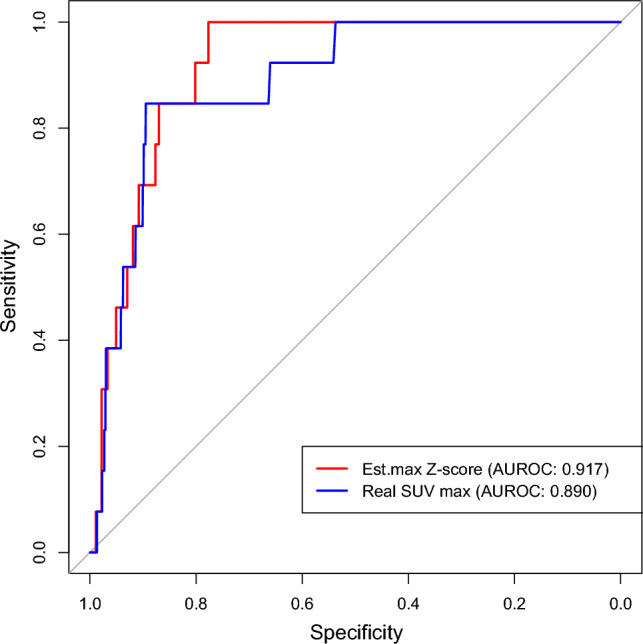
ROC curve of Z-score with SUV_max_

We found almost the same scale and same number of bins when we presented a histogram of SUV mean and estimated mean Z-score for normal cases; see Fig. 3a and c. For abnormal cases, the SUV mean and estimated mean Z-score gave a right-skewed result; see Fig. 3b and d. We also present a scatter plot of normal and abnormal cases in the same Figure. For normal cases in scatterplot, we can explain a null relationship between the SUV mean and estimated mean Z-score (Fig. 3e). By contrast, the abnormal cases explain a positive linear relationship between the two variables (Fig. 3f). Moreover, we presented the result of ROC curve of the maximum Z-score versus the SUV_max_ in Fig. 4. In Fig. 4, the AUROC value of the Z-score_max_ was higher (0.917) than that of the SUV_max_ (0.890). The Z-score map interpreted the anomaly detection approach as being higher (0.91) than the SUV_max_ (0.89). The p-value was 0.21 using Delong’s test for the two correlated ROC curves.

**Fig. 5:**
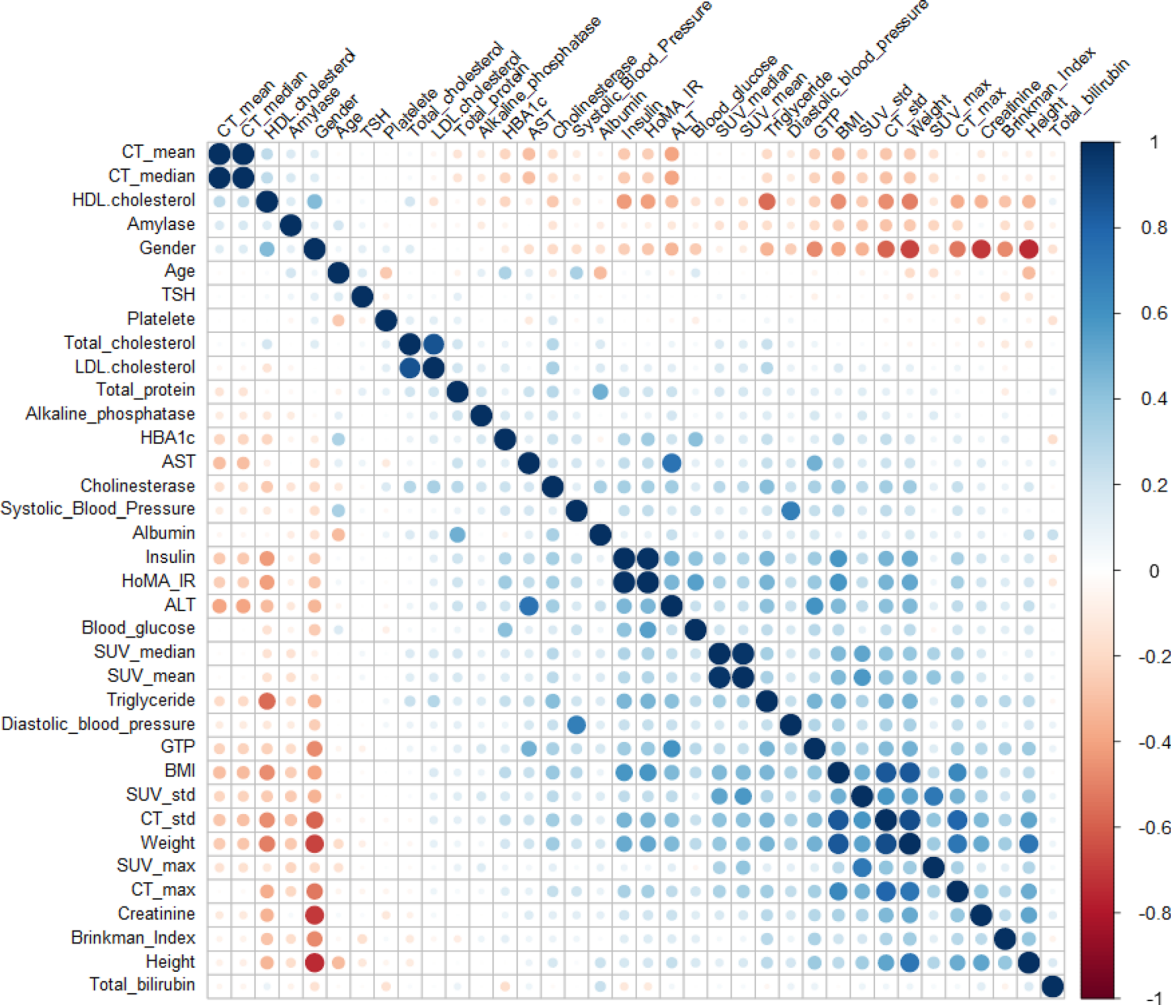
Correlation plot using heatmap

Figure 5 presents the correlation plots of the variables used in this study. BMI and weight were positively correlated with the SUV mean. For example, the correlation value of BMI and SUV mean was 0.45, while that of weight and SUV mean was 0.4. Moreover, Triglyceride (0.32) and γGPT (0.24) showed a small positive correlation among the other biochemical markers.

**Fig. 6:**
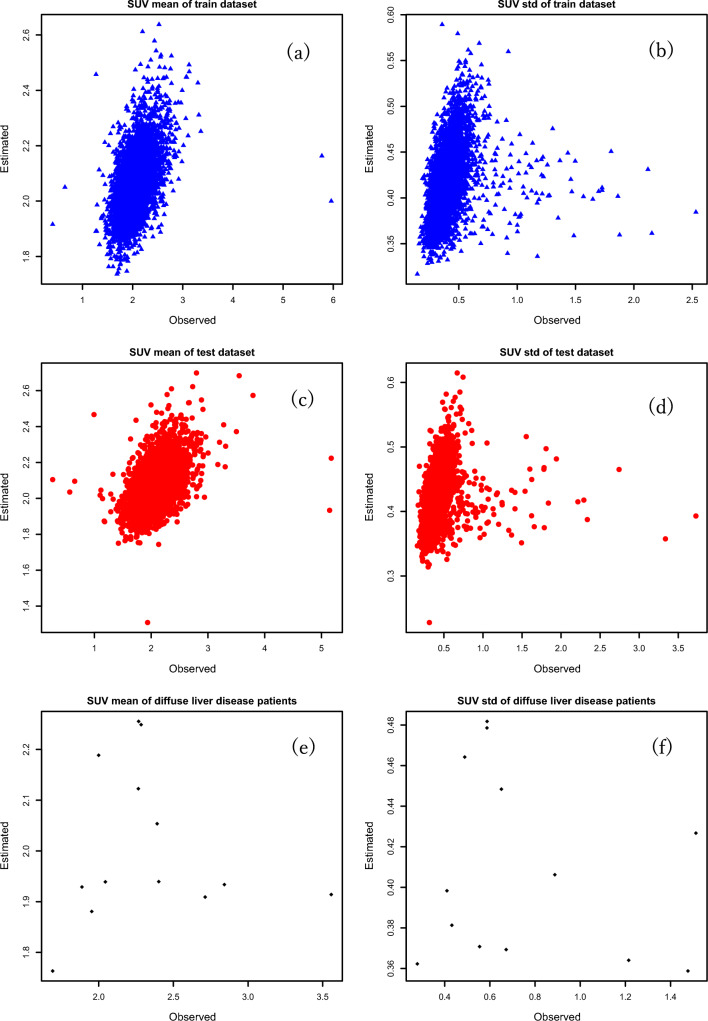
The fitted regression model is shown with the optimally selected features, as determined by the least absolute shrinkage and selection operator regression: (**a**) fitted model of the mean standardized uptake value (SUV) in the training dataset, (**b**) fitted model of SUV standard deviation in the training dataset, (**c**) fitted model of the mean SUV in the test dataset, (**d**) fitted model of SUV standard deviation in the test dataset, (**e**) fitted model of the mean SUV using the diffuse liver cases, (**f**) fitted model of SUV standard deviation using the diffuse liver cases

Finally, Figure 6 shows the LASSO linear regression model with all the selected covariates fitted to the data. We selected the covariate lists and estimated the results using the test dataset. In the model, for the threshold lambda of 0.00046, the selected covariates with their slopes (in parenthesis) are: sex (0.05), weight (0.0033), BMI (0.02), blood glucose level (0.00006), triglyceride (0.0001), total cholesterol (0.0002), GTP (0.00002), total protein (0.037), creatinine (0.017), insulin (-3.202), albumin (0.025), and cholinesterase (0.00004) for SUV mean. The intercept of the model is 0.82. Moreover, the selected covariate with its slope (in parenthesis) for SUV standard deviation in the model was weight (0.0029) for a threshold lambda of 0.000079. The intercept of the model was 0.228. The left panel of Fig. 6 shows the fitted model for the SUV mean, while the right panel shows the fitted model for the SUV standard deviation. Similarly, the top and middle rows show the models for the training and test datasets of normal cases, respectively. The bottom row shows the fitted model for abnormal cases. For normal cases, we found a positive linear relationship in the plots. Based on two-fold cross-validation, we randomly divided the entire dataset into training and test datasets.

## Discussion

This study aimed to evaluate the importance of FDG-PET SUV in identifying the liver function associated with relevant blood enzymes. Using ^18^FDG, PET/CT plays a crucial role in the treatment of various malignancies, with applications that include but are not limited to diagnosis, staging, response assessment, restaging, and prognostication. The SUV_max_ of a tumor is a crucial semi-quantitative metric used to distinguish between benign and malignant tumors. However, another method called the Z-score is used to identify abnormalities in the human body. We used the Z-score map (Eq. 1) and presented the anomaly results in Fig. 2. In Fig. 4, the findings of the per-voxel ROC analysis indicate a superior AUROC for the Z-score compared to only the SUV_max_ value in the liver region. The main advantage of using the Z-score is that it normalizes the mean and standard deviation. This normalization is effective for the ROC. Our normalization techniques increased the clarity of abnormalities. Therefore, we believe that such normalization will be a key technique for improving the diagnostic effectiveness of measuring the maximum glucose consumption of tumors. In addition, we plan to analyze the correlation between the maximum Z-score and tumor pathologies (e.g., benign vs. malignant) in a future study.

Considering the importance of the FDG-PET SUV and after analyzing it in our study, we found a significant positive correlation between the mean SUV and BMI, body weight, triglyceride, cholinesterase, and total protein and a negative correlation between the mean SUV and amylase and HDL cholesterol. Body weight and BMI have been shown to affect the transport of FDG into tissues and blood [1]. According to this study, people with a higher BMI take up more normal blood and tissue FDG than those with a lower BMI. This is probably because fat does not store much FDG during fasting; therefore, more FDG is taken up by non-fatty tissues.

In addition to Pearson correlation, we used the LASSO regression, a well-known machine learning algorithm, to filter the variables. This finding is consistent with previous studies that found a positive effect of blood glucose levels on liver FDG uptake [1]. Moreover, this is the largest study in which a large number of subjects were used to estimate the regression formula using blood test variables.

As seen in this study and previous studies, increasing blood glucose levels reduces tumor FDG absorption while boosting normal blood and tissue uptake. In addition, we presented the standard deviation of SUV uptake to create each personalized Z-score map. The covariate weight showed a positive relationship of the model with a lambda value of 7.90e-5. Our results showed the diagnostic value of the training set in the model; however, it was not significantly different from that of the testing set, which may be related to the sample size (Fig. 6).

This study has some limitations, as we did not investigate all possible variables that could affect liver FDG uptake, such as hepatic steatosis, diabetes status, abnormal lipid profile, and liver function abnormalities. Moreover, the small number of patients with abnormal findings is a drawback of this study. A few FDG-PET and blood test examinations were performed on the same day. Furthermore, we estimated only the mean and standard deviation of the entire liver, and not the voxel-wise estimation of the SUV. However, the liver is a homogenous organ; therefore, we considered the voxel-wise estimation of the SUV to be redundant. The method we introduced here is very objective and more suitable for comparing regions and backgrounds than subjective methods, such as the Deauville score.

In conclusion, the AUROC for the Z-score is a better predictor of abnormalities than the SUV_max_ value in the liver region. Covariates, such as sex, weight, BMI, blood glucose level, triglycerides, total cholesterol, GTP, total protein, creatinine, albumin, insulin, and cholinesterase are significant predictors of FDG uptake in the liver. Our future studies will include an evaluation of the predictive value of the Z-score for tumor pathology estimation.
